# Study on the Intervention Mechanism of Cryptotanshinone on Human A2780 Ovarian Cancer Cell Line Using GC-MS-Based Cellular Metabolomics

**DOI:** 10.3390/ph16060861

**Published:** 2023-06-09

**Authors:** Tong Wang, Shusheng Yin, Juan Gu, Jingjing Li, Mengmeng Zhang, Jinjun Shan, Xiao Wu, Yongming Li

**Affiliations:** 1 School of Medicine and Holistic Integrative Medicine, Jiangsu Collaborative Innovation Center of Chinese Medicinal Resources Industrialization, Nanjing University of Chinese Medicine, Nanjing 210023, China; 2 Jiangsu Key Laboratory of Pediatric Respiratory Disease, Institute of Pediatrics, Medical Metabolomics Center, Nanjing University of Chinese Medicine, Nanjing 210023, China

**Keywords:** Cryptotanshinone, A2780 cells, ovarian cancer, cell metabolomics, GC-MS

## Abstract

Cryptotanshinone (CT), an active component of the traditional Chinese medicine *Salvia miltiorrhiza* Bunge, exhibits a wide range of biological and pharmacological activities. Although the anticancer activity of CT is well known, the knowledge of its effect on the regulation of cancer cell metabolism is relatively new. The present study investigated the anticancer mechanism of CT in ovarian cancer with a focus on cancer metabolism. CCK8 assays, apoptosis assays, and cell cycle assays were conducted to reveal the growth-suppressive effect of CT on ovarian cancer A2780 cells. To explore the potential underlying mechanisms of CT, the changes in endogenous metabolites in A2780 cells before and after CT intervention were investigated using the gas chromatography–mass spectrometry (GC-MS) approach. A total of 28 important potential biomarkers underwent significant changes, mainly involving aminoacyl-tRNA biosynthesis, energy metabolism, and other pathways. Changes in the ATP and amino acid contents were verified with in vitro and in vivo experiments. Our results indicate that CT may exert an anti-ovarian cancer effect by inhibiting ATP production, promoting the protein catabolic process, and inhibiting protein synthesis, which may lead to cell cycle arrest and apoptosis.

## 1. Introduction

Ovarian cancer is considered one of the most fatal cancers among malignant tumors [1]. Difficulties in the early diagnosis of ovarian cancer due to a lack of effective screening strategies lead to high morbidity and mortality. The estimated numbers of new ovarian cancer cases and deaths in China were 55,342 and 37,519, accounting for 17.63% and 18.10% of global cases, respectively [2]. Surgery and chemotherapy are currently the mainstream treatment options for ovarian cancer. However, the prognosis of ovarian cancer remains very poor due to severe toxicity and the development of drug resistance against prevailing chemotherapy drugs [3]. Therefore, the development of more potent anticancer drugs with fewer side effects and a novel mechanism of action is extremely important. 

Natural products have played and continue to play a significant role in the treatment of various ailments, including cancer. It is estimated that approximately 60% of all anticancer drugs available on the market at present are derived from natural products [4,5]. Cryptotanshinone (CT) is a naturally occurring bioactive molecule of the quinine family that has received increased attention from the scientific community due to its impressive antitumor activity against a wide range of human cancers. The potential molecular mechanisms involved in the anticancer activity of CT include the inhibition of cell proliferation, migration, and invasion; the induction of apoptosis; the modulation of estrogen and androgen receptors; the reversion of drug resistance; and the inhibition of aberrantly activated cell survival signaling pathways [6,7,8,9]. Apart from its anticancer activity as monotherapy, CT has been shown to improve the anticancer activity of various clinical anticancer drugs in combination therapy [10]. Recently, CT has been reported to inhibit the growth of breast and ovarian cancer by regulating glucose metabolism [11,12]. However, the detailed mechanism of this metabolic regulation remains unknown. 

Aberrant metabolism is one of the hallmarks of cancer cells, and the metabolic pathways are important targets of cancer therapy [13]. Gas chromatography–mass spectrometry (GC-MS) has been extensively used in metabolomics and plays a significant role in the treatment of cancers [14]. In this study, we aimed to investigate the alterations in the metabolic profiles of ovarian cancer cells after CT intervention and elucidate the mechanism of CT in ovarian cancer cells by identifying biomarkers and the corresponding metabolic pathways. The anticancer activity of CT was evaluated in A2780 ovarian cancer cells and a xenograft tumor model. This investigation provides ideas for studying antitumor mechanisms in traditional Chinese medicine.

## 2. Results

### 2.1. Effect of CT on Cell Viability Assay

**Figure 1:**
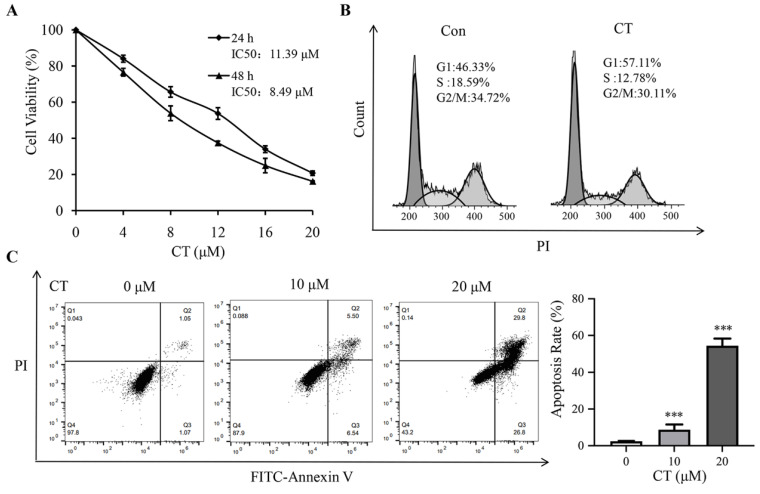
CT inhibited growth of A2780 cells, promoted G1-phase cell cycle arrest, and induced apoptosis. (**A**) Cell viability of the A2780 cells untreated or treated with various concentrations of CT for 24 and 48 h. (**B**) Flow cytometry analysis of cell cycle profile of A2780 cells treated with CT (10 μM) for 24 h. The percentage of cells in the G1, S, or G2/M phases of the cell cycle is indicated. (**C**) Apoptotic rate of A2780 cells untreated or treated with CT (10 and 20 μM) for 24 h. Differences between two groups were analyzed using Student’s *t*-test. *** *p* < 0.001 compared with the control group. Data are expressed as mean ± SD (*n* = 3).

The CCK-8 results show that CT significantly inhibited the survival of A2780 cells in a dose-dependent manner (Figure 1A). The IC_50_ values of the A2780 cells treated with CT were 11.39 and 8.49 μM after 24 and 48 h, respectively. Based on the IC_50_ values and the living cell metabolites, a 10 μM CT dose and 24 h drug exposure time were selected for further metabolomics research.

### 2.2. Analysis of Cell Cycle Profiles and Apoptosis

Flow cytometry was employed to detect the apoptosis and cell cycle profiles of the control and CT treatment groups. Compared with the control group, the percentage of cells in the G1 phase in the CT treatment group significantly increased with a subsequent decrease in the S and G2/M phases (Figure 1B). Moreover, CT significantly induced apoptosis in A2780 cells in a dose-dependent fashion (Figure 1C).

### 2.3. GC-MS Cell Metabolomics Analysis

#### 2.3.1. Discriminant Analysis of Model

**Figure 2:**
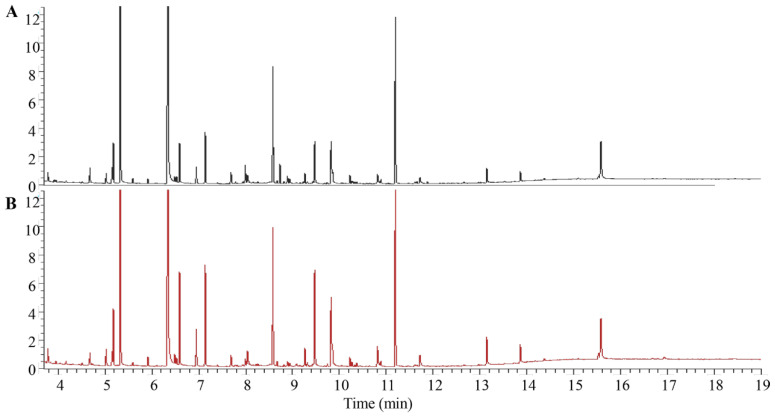
GC-MS total ion chromatograms of the A2780 cells. (**A**) Control group. (**B**) CT-treated group.

**Figure 3:**
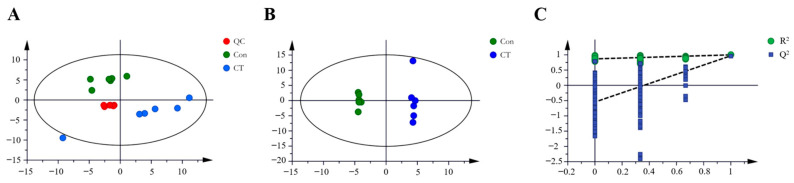
Multivariate analysis derived from total ion chromatograms (*n* = 6). (**A**) Principal component analysis (PCA) score plot of QC, Con (control group), and CT (CT-treated group). (**B**) OPLS-DA score plots. (**C**) OPLS-DA validation plot: R^2^ = 0.869; Q^2^ = −0.502.

A total ion flow chromatogram of metabolites in both untreated and treated groups analyzed using GC-MS is shown in Figure 2. After normalization and standardization, PCA was performed using SIMCA software (Figure 3A). The QC samples were concentrated at the origin and clustered tightly, indicating that the method was stable and repeatable. Additionally, the CT treatment group and the blank group were significantly separated, indicating that the cell metabolites in the two groups were significantly different from each other.

Furthermore, orthogonal partial least squares discriminant analysis (OPLS-DA) was used to filter the irrelevant signals and obtain the OPLS-DA model (Figure 3B). The values of the R^2^Xcum, R^2^Ycum, and Q^2^Ycum fitting parameters were 0.694, 0.999, and 0.969, respectively, suggesting that the model had good stability and high predictability. To avoid over-fitting, cross-validation was used to test the quality of the model, and 200 permutation tests were performed on the model. After cross-validation, the intercepts of R^2^ and Q^2^ with the Y-axis were 0.869 and −0.502, respectively, indicating that the model was not over-fitting (Figure 3C).

#### 2.3.2. Identification of Differential Metabolites

**Figure 4:**
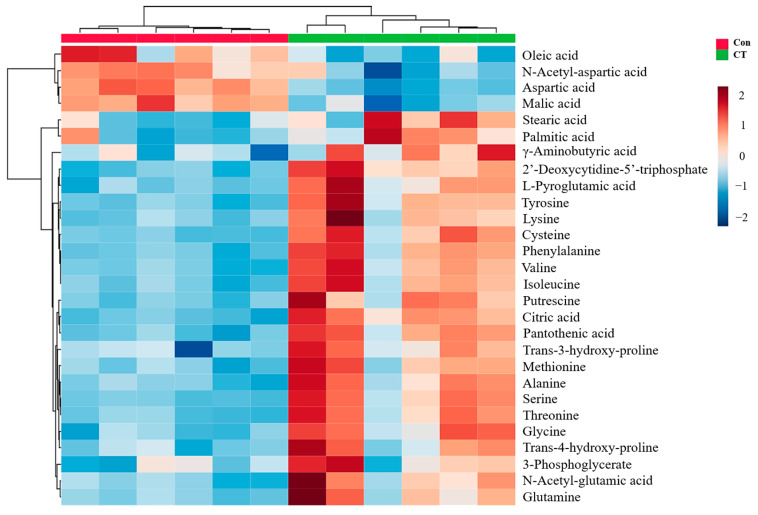
Heat map of different cell metabolite distributions in control and CT-treated groups (*n* = 6).

Table 1 shows the *p*-value and VIP value calculated with SIMCA. A total of 28 different metabolites were identified according to *p* < 0.05 and VIP > 1. Aspartic acid, malic acid, N-acetyl-aspartic acid, and oleic acid were down-regulated via CT. Oleic acid, citric acid, deoxidization cytidine triphosphate, pantothenic acid, cysteine, serine, phenylalanine, tyrosine, threonine, valine, isoleucine, glutamic acid, glycine, putrescine, alanine, methionine, lysine, 3-hydroxy proline, N-acetyl-glutamic acid and glutaminases, 4-hydroxyproline, stearic acid, γ-aminobutyric acid, palmitic acid, and 3-phosphoglyceric acid were up-regulated. Figure 4 presents the heat map of the difference between the metabolite distributions of the blank group and the treatment group. Red represents the up-regulation of intracellular metabolite content, and blue represents down-regulation. The results show that there were significant differences in the endogenous metabolites between the cells treated with CT and the blank group.

#### 2.3.3. Metabolic Pathway Analysis

**Figure 5:**
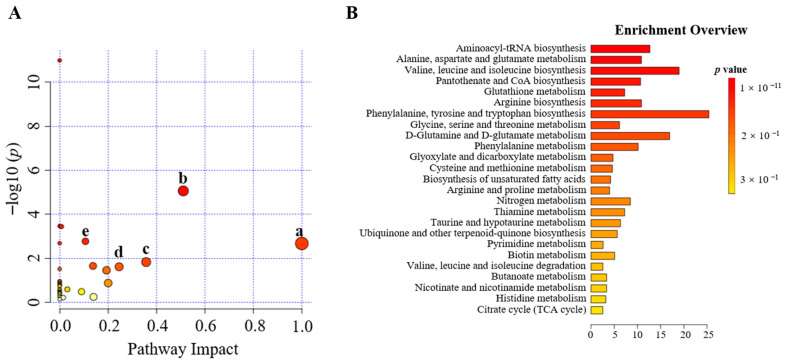
Score plot for metabolic pathway analysis. (**A**) Pathway analysis of differential metabolites between control group and CT-treated group. (a) Phenylalanine, tyrosine, and tryptophan biosynthesis, (b) alanine, aspartate, and glutamate metabolism, (c) phenylalanine metabolism, (d) glycine, serine, and threonine metabolism, (e) glutathione metabolism. (**B**) Overview of the pathway enrichment.

**Figure 6:**
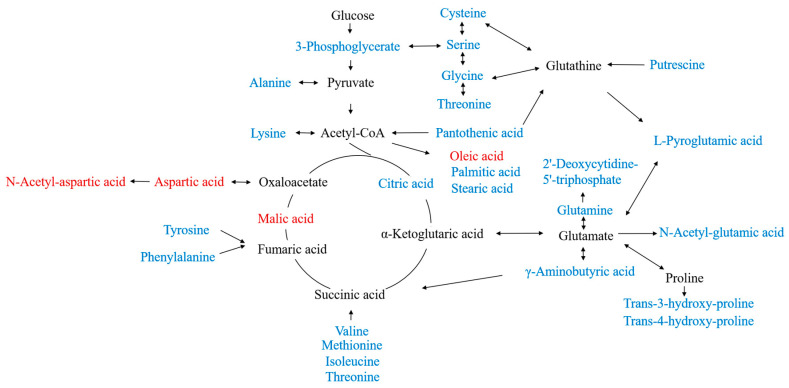
Schematic diagram of the network of metabolic pathways associated with different metabolites. Red indicates downward adjustment; blue indicates upward adjustment.

We imported the 28 differential metabolites into MetaboAnalyst 5.0 for online pathway analysis to study the changes in metabolic pathways after CT intervention [15,16]. The circles in the metabolic pathway diagram represent contribution values, and the larger the circle, the greater the contribution (Figure 5A). The metabolic pathway analysis found that CT interfered with the biosynthesis as well as the metabolism of various molecules. As shown in Figure 5B, CT interfered with the biosynthesis of aminoacyl-tRNA, pantothenic acid, CoA, arginine, valine, leucine, isoleucine, phenylalanine, tyrosine, tryptophan, and unsaturated fatty acids in A2780 cells. Similarly, CT also interfered with alanine, glutamic acid, aspartic acid, glycine, serine, threonine, glutamine, glutamate, phenylalanine, glyoxylic acid, dicarboxylic acid, cysteine, methionine, and glutathione metabolism. These pathways may be closely related to the therapeutic function of CT in ovarian cancer. To analyze the relationships between different metabolites, we combined the KEGG database with the related literature to establish a metabolic network diagram between 28 CT biomarkers and metabolic pathways (Figure 6). The biomarkers of CT intervention in A2780 cells are mainly related to amino acid metabolism, and different metabolites participate in the tricarboxylic acid cycle (TCA) through the connections between intermediate products and acetyl-CoA.

### 2.4. Determination of Intracellular ATP and Amino Acid Contents

**Figure 7:**
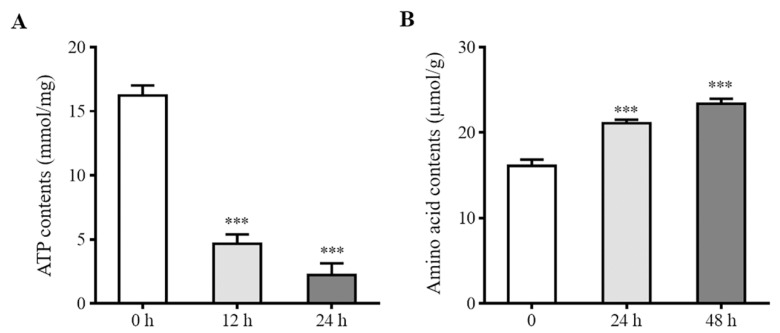
Effects of CT on intracellular ATP and amino acid contents. (**A**) ATP contents in A2780 cells in the absence or presence of CT (10 μM) for 12 and 24 h. (**B**) Amino acid contents in A2780 cells in the absence or presence of CT (10 μM) for 24 and 48 h. Differences between two groups were analyzed using Student’s *t*-test. *** *p* < 0.001 compared with the control group. Data are expressed as mean ± SD (*n* = 3).

The intracellular ATP contents of the A2780 cells were measured to assess the effect of CT on energy metabolism. It was shown that ATP decreased significantly after treatment with CT for 12 and 24 h. The ATP content was 16.23 nmol/mg in the blank group, while the ATP contents in the treated group were 4.66 nmol/mg and 2.23 nmol/mg after 12 and 24 h, respectively (Figure 7A).

In addition, we compared the amino acid contents in the cells to preliminarily test the levels of amino acid metabolism. Compared with the control group, the amino acid contents in the CT-treated group were significantly up-regulated (Figure 7B).

### 2.5. Effect of CT on A2780 Xenograft Tumor Growth

**Figure 8:**
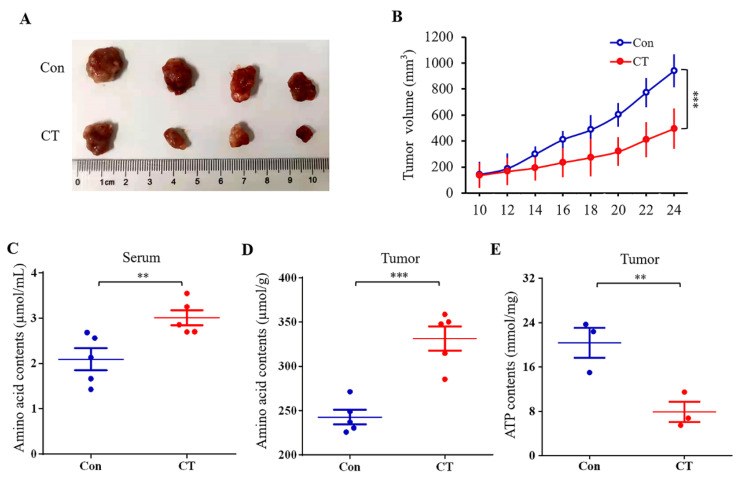
Effects of CT on xenograft tumor growth and the amino acid and ATP contents. (**A**) Representative image of tumor growth in control and CT groups (*n* = 4). (**B**) Tumor growth curves in control and CT groups (*n* = 4). (**C**) The effect of CT on amino acid contents in serum (*n* = 5). (**D**) The effect of CT on amino acid contents in tumor tissue (*n* = 5). (**E**) The effect of CT on ATP contents in tumor tissue (*n* = 3). Data are expressed as mean ± SD. Differences between two groups were analyzed using Student’s *t*-test. ** *p* < 0.01 and *** *p* < 0.001 compared with the control group.

Compared with the mean xenograft tumor volume in the blank groups, the volume of the transplanted tumor in the experimental group decreased significantly (Figure 8A,B). We discovered that CT significantly reduced the volume and weight of subcutaneous xenograft tumors. The amino acid contents in both serum and tissue increased in the CT groups (Figure 8C,D), whereas the ATP contents decreased (Figure 8E).

## 3. Discussion

The KEGG pathway enrichment analysis showed that CT had the most significant effects on the aminoacyl-tRNA biosynthetic pathway, suggesting that CT caused dysfunction in protein syntheses. It was demonstrated that CT attenuated tumor growth by activating the AMP-activated protein kinase (AMPK) signaling pathway. The activation of the AMPK signaling pathway leads to the suppression of mTOR activity, which is responsible for the inhibition of protein synthesis [17,18]. Altered aminoacyl-tRNA biosynthesis may cause oxidative stress, which may ultimately sabotage the cellular and metabolic activities of tumor cells [19]. The disordered glutathione metabolism in the CT treatment group may be related to the increases in the intracellular glycine, cysteine, putrescine, and pyroglutamate contents. It is known that glutathione is involved in redox regulation and plays an important role in the antioxidant defense system and the oxidation-dependent regulation system [20]. It was reported that serine promotes the survival and proliferation of cancer cells [21] and regulates the cell anti-oxidant system [22]. The increase in the serine content in the cells of the treatment group suggests that CT may disrupt the antioxidant system in A2780 cells.

The results show that the malic acid, aspartic acid, and acetyl-aspartic acid contents in the cells of the treatment group were down-regulated. Citric acid and malic acid are important components of the TCA cycle. It has been revealed that the malate–aspartate shuttle promotes the transfer of NADH from the cytoplasm to mitochondria and oxidative phosphorylation, which is crucial for maintaining a high glycolysis rate and supporting the rapid growth of tumor cells [23]. CT treatment was found to affect branched-chain amino acid metabolites, such as valine and isoleucine, which are not only involved in protein synthesis, but are also related to energy metabolism [24]. In the treatment group, it was found that the metabolic pathways of glyoxylic acid and dicarboxylic acid, which are closely related to energy metabolism, were changed and may be related to the differentiation ability of tumor cells [23]. Pantothenic acid, as an intermediate product of the TCA cycle, participates in the synthesis of acetyl-CoA [25]. Our results show that the intracellular pantothenic acid content increased after CT treatment, which indicates that the cell energy metabolism was out of balance. The glycine, serine, and threonine contents increased in cells treated with CT. It is known that glycine, serine, and threonine provide energy via the conversion of pyruvate to acetyl-CoA to enter the TCA cycle [26]. The changes in the metabolites in the above pathways suggest that CT may affect the energy metabolism in A2780 cells, causing the imbalance between cell energy metabolism and cell activity inhibition.

The experimental results show that the contents of aspartic acid and glutamic acid, which are the raw materials in the synthetic process of pyrimidine, increased in cells treated with CT [27]. The increase in deoxycytidine triphosphate in the treatment group cells suggests that CT may affect the nucleotide metabolism in A2780 cells.

To investigate the effects of CT on energy metabolism and amino acid metabolism, we further detected the ATP and amino acid contents in vitro and in vivo. CT treatment led to a significant reduction in intracellular ATP levels, which was also confirmed in vivo. Changes in the ATP contents were consistent with the results obtained in the metabolomics study. Numerous studies have confirmed that amino acid metabolism is deregulated in many cancers [28,29,30]. Amino acids are potential biomarkers of ovarian cancer and are related to tumor growth, playing an important role in cancerogenesis [31,32]. A previous study revealed abnormalities in the serum-free amino acid profiles of ovarian cancer patients [33]. In our study, CT increased amino acid contents in A2780 cells as well as in the serum and tumor tissue of the xenograft models. This observation is consistent with the aforementioned report.

We observed increased levels of glutamine in the CT group compared with the control group. Glutamine plays a vital role in the TCA cycle by providing a carbon source [34]. It can be transformed into α-ketoglutarate, which is a TCA cycle intermediate for energy recycling. The up-regulation of glutamine after CT treatment may be attributed to the inhibition of glutamine metabolism and the TCA cycle. Glutamine metabolism is involved in both glycolysis and the TCA cycle. It has been demonstrated that CT inhibited glycolysis via the STAT3/SIRT3 signaling pathway in ovarian cancer cells [12]. The inhibition of glycolysis may lead to a compensatory increase in glutamine [35].

The down-regulation of the intracellular oleic acid content and the up-regulation of the palmitic acid and stearic acid content suggest that CT may affect the lipid metabolism in A2780 cells.

CT treatment increased the content of the neurotransmitter metabolite γ-aminobutyric acid in A2780 cells and decreased the content of N-acetyl-aspartic acid. Moreover, the increases in the phenylalanine and tyrosine contents in the treatment group may be related to neurotransmitter production [36]. Neurotransmitters can play a key role in the regulation of tumor cells and can influence the formation and development of tumors. Experimental data have shown that γ-aminobutyric acid receptors are widely expressed in a variety of cancer tissues and cancer cells and are related to the progression of ovarian cancer [1,37,38]. N-acetyl-aspartic acid is considered as a marker for the diagnosis of complex cystic masses such as benign teratoma, endometriosis, and tubo-ovarian abscesses [39]. Furthermore, its ratio to choline has proved to be a reliable biomarker for differentiating between borderline and malignant epithelial ovarian tumors [40]. Therefore, the antitumor effect of CT may be related to neurotransmitter disorders, and neurotransmitters such as γ-aminobutyric acid may offer potential as biomarkers.

## 4. Materials and Methods

### 4.1. Materials

High-purity (≧98%) CT, Cell counting Kit-8 (CCK8), and the Annexin V-FITC Apoptosis Detection Kit were purchased from Dalian Meilun Biotechnology Co., Ltd. (Dalian, China). DMEM medium (high glucose) was purchased from Hyclone Company (Hyclone, Logan, UT, USA). Fetal bovine serum (FBS) was obtained from Gibco (Grand Island, NY, USA). The BCA Protein Assay Kit and propidium iodide were purchased from Shanghai Yeasen Biotechnology Co., Ltd. (Shanghai, China). RNaseA was obtained from Thermo Fisher Scientific Inc. (Waltham, MA, USA). The ATP Assay Kit was purchased from Shanghai Biyuntian Biotechnology Co., Ltd. (Shanghai, China). Dimethyl sulfoxide (DMSO) was purchased from Beijing Solarbio Science and Technology Co., Ltd. (Beijing, China). Myristic acid-1,2-^13^C_2_, N, O-Bis (trimethylsilyl) trifluoroacetamide (BSTFA), trimethylchlorosilane (TMCS), pyridine, and methoxamine were purchased from Sigma-Aldrich (St. Louis, MO, USA).

### 4.2. Cell Lines

The A2780 Human ovarian cancer cell line was obtained from the European Collection of Authenticated Cell Cultures (ECACC). A2780 cells were cultured in DMEM supplemented with 10% FBS and cultured at 37 °C with 5% CO_2_. 

### 4.3. Cell Proliferation Assay

The A2780 cells were incubated in 96-well plates at 1 × 10^4^ cells/well with 100 µL of DMEM. Following treatment with 4, 8, 12, 16, and 20 μM of CT for 24 and 48 h, 10 μL of CCK-8 solution was added to each well, and they were incubated at 37 °C for 1 h under dark conditions. Absorbance was measured at 450 nm with a microplate reader, and the cell survival rate and half inhibitory concentration (IC_50_) were calculated.

### 4.4. Apoptosis and Cell Cycle Analysis

Apoptosis assays were conducted as follows: the cells were seeded into 6-well tissue culture plates and treated with 0 and 10 μM of a CT-medicated medium for 24 h. The cells were harvested and resuspended in a binding buffer. Then, Annexin V-FITC and PI were added, and the cells were incubated at room temperature for 15 min in the dark according to the instructions. The cells were analyzed using flow cytometry after 200-mesh screening.

The cell cycle analysis was conducted as follows: A2780 cells were treated with 0 and 10 μM of CT for 24 h. The cells were collected and resuspended in 300 μL of PBS. An amount of 700 µL of precooled absolute ethanol was slowly added to fix the cells at 4 °C for 3 h. After the samples were washed twice with PBS, 500 μL of a precooled PBS-Pi-RNaseA mixture (PI: 50 μg/mL; RNase A: 20 μg/mL) was added to each sample, which was then resuspended and incubated at room temperature for 15 min in the dark. The cells were analyzed using flow cytometry after 200-mesh screening.

### 4.5. GC-MS Sample Preparation

A2780 cells in the logarithmic growth phase were seeded in 6-well plates (5×10^5^ cells/well) and divided into blank groups and experimental groups. The cells were treated with DMSO or CT (10 μM) for 24 h. Six parallel wells were set up for each group. Then, the supernatant was discarded, and the cells were washed 3 times with pre-chilled PBS. Cellular metabolism was quenched by adding liquid nitrogen, and 300 μL of pure water was added for storage at −80 °C. Following 3 freeze–thaw cycles (freezing at −80 °C for 60 min and melting at 37 °C for 30 min), 20 μL of each sample was used to measure the protein concentration with a BCA kit. An amount of 400 μL of methanol solution (containing 4 μg of myristic acid-1,2-^13^C_2_ as an internal standard) was added to the remaining samples, and they were sonicated for 1 min. QC samples were prepared by vortex mixing 30 μL samples. The remaining samples and QC samples were centrifuged for 2 min (14,000 rpm) at 4 °C. An amount of 300 μL of each supernatant was placed into a new tube and evaporated in a centrifugal concentrator, 30 μL of a 40 mg/mL solution of methoxamine hydrochloride in pyridine was added, and the mixture was vortexed for 5 min. After oscillating in a constant-temperature oscillator for 1.5 h (300 rpm) at 30 °C, 30 μL of BSTFA solution containing 1% TMCS was added to the supernatant, and the mixture continued oscillating for 10 min at 4 °C (18,000 rpm). An amount of 50 μL of supernatant was removed and analyzed using GC-MS. The QC samples were analyzed before sample analysis and after every 4 samples were analyzed to investigate the stability of the analysis system.

### 4.6. GC-MS Analysis Conditions

GC-MS was performed using a Thermo Trace 1310 GC-MS system (Thermo Fisher Scientific, USA). First, 1 μL of each sample was injected and separated with a TG-5MS GC column (30 m × 0.25 mm, 0.25 μm; Thermo Fisher, USA) with a split ratio of 20:1. Helium (purity > 99.99%) was used as the carrier gas and maintained at a constant flow rate of 1.2 mL/min. The initial oven temperature was held at 60 °C for 1 min, and then the temperature was increased to 320 °C at a rate of 20 °C/min, and finally maintained at 320 °C for 5 min. The electron energy was 70 eV. The transmission line and ion source temperatures were 250 and 280 °C, respectively. MS data were acquired in full-scan mode with a mass range of 50–500 *m*/*z* and the collection time was 3.5–19 min.

### 4.7. Data Processing and Analysis

Collected GC/MS data were converted using an ABF Converter, and the MS-DIAL software was employed to identify and align the peaks. The FiehnLib database was used to match the chromatographic peaks with metabolites, and metabolites with a similarity greater than 80% were further matched with fragment ion peaks using the NIST library. The peak area was corrected with the internal standard peak area and protein concentration. SIMCA 14.0 software was applied to perform unsupervised principal component analysis (PCA) and supervised orthogonal partial least squares discriminant analysis (OPLS-DA) combined with orthogonal noise filtering (OSC). The data were normalized, and the different metabolites were screened according to VIP values (VIP > 1) and *p*-values (*p* < 0.05). The metabolites, peak values, and other information were imported into the MetaboAnalyst 5.0 database for cluster analysis. After Pareto scaling, the Ward clustering algorithm was used to draw heat maps, and the hypergeometric test was used for path enrichment analysis. Metabolic pathways were screened according to *p*-values (*p* < 0.05).

### 4.8. Determination of Intracellular ATP Contents

A2780 cells were seeded in 6-well plates and treated with 0 and 10 μM of CT for 12 and 24 h, respectively. An amount of 200 μL of lysate was added to each well to lysate the cells. Then, the supernatants were centrifuged for 5 min at 4 °C (12,000 rpm). The ATP concentrations were calculated with the ATP assay kit (Beyotime Biotechnology Co., Ltd., Shanghai, China) according to the standard curve, and protein concentrations were determined using the BCA kit (Beyotime Biotechnology Co., Ltd., Shanghai, China).

### 4.9. Determination of Intracellular Amino Acid Contents

A2780 cells were treated with 0 and 10 μM of CT for 24 and 48 h. The cells were harvested, and amino acid contents were determined using a micro amino acid content assay kit (Beijing Solarbio Science and Technology Co., Ltd., Beijing, China). The amino acid contents were normalized to the corresponding protein concentrations.

### 4.10. Mouse Xenograft Models

Female BALB/c nude mice (5 weeks old; 16–18 g) were purchased from Jiangsu GemPharmatech Biotechnology Co., Ltd. (Nanjing, China). All the mice were kept in an SPF-level laboratory animal room and fed with sterilized food and water at the Animal Center of the Nanjing University of Chinese Medicine. The animal studies were approved by the Committee on Animal Research and Ethics of the Nanjing University of Chinese Medicine. All animal procedures were carried out following the National Institutes of Health Guide for the Care and Use of Laboratory Animals.

A2780 cells in the logarithmic growth stage were selected to prepare cell suspensions with 2 × 10^7^ cells/mL concentrations. To establish the A2780 human ovarian cancer xenograft tumor model, each nude mouse was subcutaneously inoculated with 100 μL of cell suspension in the left anterior axillary region. Tumor growth was observed 10 days after inoculation. The A2780 tumor-bearing mouse model was successfully established until the diameter of the tumor volume reached 2 mm. The mice were randomly divided into blank and experimental groups and received intraperitoneal injections containing 10 mg/kg of CT or normal saline every 3 days.

The long diameters (a) and short diameters (b) of the tumors were measured every 2 days using vernier calipers. The tumor volumes were calculated using the following formula: V (mm^3^) = 3.14ab^2^/6. Tumor growth was observed continuously for 2 weeks, and tumor growth curves were drawn.

After 2 weeks of inoculation, all nude mice were sacrificed, and blood was collected. The tumors were stripped and weighed, and 1 mL of blood was collected from the orbital veins. Serum was separated and stored at −80 °C. The amino acid contents in serum and tumor tissue were determined according to the instructions of the amino acid content assay kit. The ATP content in tumor tissue was determined according to the instructions of the ATP assay kit.

## 5. Conclusions

In the present study, the inhibitory effect of CT was evaluated in A2780 human ovarian cancer cells and a xenograft tumor model. CT significantly induced cell cycle arrest and apoptosis in A2780 cells in vitro and inhibited tumor growth in vivo. The antitumor mechanism of CT was further clarified using GC-MS-based cellular metabolomics. The significantly altered metabolites were mainly associated with amino acid metabolism and energy metabolism, which are involved in the aminoacyl-tRNA biosynthesis pathway; alanine, aspartate, and glutamate metabolism; and pantothenate and CoA biosynthesis. Consistent with the metabolomics results, CT increased the amino acid contents and decreased the ATP contents in A2780 cells and xenograft tumors. This study reveals new insights into the underlying anticancer mechanism of CT and provides references for the discovery of ovarian cancer biomarkers.

## Figures and Tables

**Table 1:** Different metabolites in cells after CT treatment.

No.	Name	Average Rt (min)	*p* Value	VIP	Fold Change	Trend
1	Aspartic acid	7.989	0.0000	1.68	0.40	↓ ***
2	Citric acid	9.746	0.0000	1.60	2.80	↑ ***
3	2′-Deoxycytidine -5’-triphosphate	7.765	0.0001	1.58	3.03	↑ ***
4	Pantothenic acid	10.642	0.0001	1.56	1.67	↑ ***
5	Malic acid	7.781	0.0001	1.53	0.65	↓ ***
6	Cysteine	8.224	0.0002	1.52	2.17	↑ ***
7	Serine	6.946	0.0003	1.50	2.21	↑ ***
8	Phenylalanine	8.676	0.0003	1.52	2.01	↑ ***
9	Valine	5.914	0.0003	1.50	1.78	↑ ***
10	Tyrosine	10.367	0.0004	1.50	2.35	↑ ***
11	Threonine	6.495	0.0005	1.46	1.76	↑ ***
12	Isoleucine	6.484	0.0006	1.47	1.87	↑ ***
13	L-Pyroglutamic acid	8.034	0.0009	1.47	1.83	↑ ***
14	Glycine	5.150	0.0009	1.45	1.58	↑ ***
15	Putrescine	9.314	0.0011	1.40	2.01	↑ **
16	Alanine	5.024	0.0013	1.41	1.61	↑ **
17	N-Acetyl-aspartic acid	8.819	0.0015	1.40	0.74	↓ **
18	Methionine	8.000	0.0023	1.37	1.67	↑ **
19	Lysine	10.265	0.0036	1.37	2.08	↑ **
20	Trans-3-hydroxy-proline	8.018	0.0043	1.30	2.31	↑ **
21	N-Acetyl-glutamic acid	9.397	0.0050	1.30	1.86	↑ **
22	Glutamine	9.074	0.0065	1.28	2.81	↑ **
23	Oleic acid	11.619	0.0067	1.27	0.77	↓ **
24	Trans-4-hydroxy-proline	8.040	0.0107	1.22	1.42	↑ *
25	Stearic acid	11.718	0.0174	1.14	1.10	↑ *
26	γ-Aminobutyric acid	3.781	0.0216	1.15	1.06	↑ *
27	Palmitic acid	10.813	0.0262	1.07	1.10	↑ *
28	3-Phosphoglycerate	9.688	0.0455	1.05	1.32	↑ *

Note: * *p* < 0.05, ** *p* < 0.01, and *** *p* < 0.001 compared with the control group. ↑: upward adjustment; ↓: downward adjustment.

## Data Availability

Data is contained within the article.
